# Airway First: A Retrospective Study of Tracheotomy-Primed Sclerotherapy for High-Risk Cervicofacial Venous Malformations

**DOI:** 10.3390/jcm14176154

**Published:** 2025-08-30

**Authors:** Xuan Jiang, Li Hu, Xi Yang, Yunbo Jin, Hui Chen, Xiaoxi Lin

**Affiliations:** 1 Department of Plastic and Reconstructive Surgery, Shanghai Ninth People’s Hospital, Shanghai Jiao Tong University School of Medicine, Shanghai 200011, China; jiangx0110@163.com (X.J.); huli89@foxmail.com (L.H.); docyang9h@163.com (X.Y.); docjinyunbo@163.com (Y.J.); 2 Department of Laser and Aesthetic Medicine, Shanghai Ninth People’s Hospital, Shanghai Jiao Tong University School of Medicine, Shanghai 200011, China

**Keywords:** tracheotomy, sclerotherapy, venous malformations, upper respiratory tract, ACSTS strategy

## Abstract

**Objectives:** This study assesses the efficacy of tracheotomy-primed sclerotherapy in craniofacial venous malformations (VMs), establishes evidence-based airway intervention criteria, and develops site-specific safety protocols to optimize treatment timing and safety in cases with upper airway compromise. **Methods:** We retrospectively collected the clinical data of 35 patients treated by our center between January 2008 and November 2024, who were diagnosed with cervicofacial VMs involving the upper respiratory tract. All patients underwent direct tracheotomy or tracheotomy after sclerotherapy for lesions located in the anterior cervical area. Sclerotherapy was performed under fluoroscopy or laryngoscopy after tracheotomy. **Results:** 35 patients underwent 225 sclerotherapy sessions. Nineteen patients underwent tracheotomy directly, and sixteen patients received sclerotherapy at the anterior cervical area before tracheotomy. All patients presented improvement according to magnetic resonance imaging (MRI) findings, and 94.29% (33/35) of patients reported improvement in clinical presentations. All patients experienced improvement in quality of life (QoL). No major complications occurred. Decannulation was successfully performed in all 35 patients after finishing sclerotherapy. **Conclusions:** Tracheotomy followed by sclerotherapy is safe and effective for VMs involving the upper respiratory tract. This is necessary for patients with lesions involving the laryngopharyngeal region, tongue base, and bilateral pharyngeal walls. In high-risk prophylactic tracheostomy candidates, anterior cervical sclerotherapy–tracheostomy–sclerotherapy (ACSTS) is an effective strategy for managing airway obstruction.

## 1. Introduction

VMs are one of the most common benign vascular anomalies, with approximately 40% of cases occurring in the head and neck region [1]. The lesions grow in proportion to the body, demonstrating lifelong development, and do not regress spontaneously [2]. Extensive head and neck venous malformations are not only disfiguring, but also accompanied by functional disorders. They may result in swallowing, speech, and airway problems, and may even lead to death due to bleeding and suffocation, when the respiratory tract is involved [3].

Sclerotherapy has been considered the first-line treatment for head and neck VMs [4]. Multiple agents are available as sclerosants when performing sclerotherapy, including absolute ethanol, sotradecol, bleomycin, polidocanol, etc. [5]. Jin et al. treated VMs with absolute ethanol combined with pingyangmycin, achieving significant improvement in 95% (114/196) of patients [6]. Subsequently, Yang et al. applied bleomycin–polidocanol foam sclerotherapy, with all 55 patients (100%) reporting symptom relief and an overall excellent or good response rate of 94.6% [7]. As several sclerosing agents have shown comparable efficacy and complication profiles, systematic reviews in this area continue to emerge [8]. However, percutaneous sclerotherapy is frequently associated with significant airway swelling for head and neck VMs involving the respiratory tract, which could lead to life-threatening airway obstruction. An emergency tracheotomy was often required for airway maintenance under such circumstances [9]. Therefore, we proposed that prophylactic tracheotomy combined with sclerotherapy may be a safe and effective treatment for VMs involving the respiratory tract.

This study aims to critically evaluate the effectiveness of post-tracheostomy sclerotherapy in craniofacial VM patients while establishing evidence-based tracheostomy criteria and developing safe, anatomically stratified management protocols for VM cases with upper airway compromise, ultimately bridging current knowledge gaps in intervention timing and procedural safety.

## 2. Materials and Methods

### 2.1. Study Design

Between September 2008 and November 2024, the charts of 35 patients who suffered from extensive head and neck VMs with upper respiratory tract involvement and underwent tracheotomy combined with sclerotherapy in our vascular anomaly center were reviewed. The demographics and lesion characteristics are summarized in Table 1. Extensive VMs were defined as lesions involving more than three anatomic sites. All VMs were diagnosed according to the criteria of the International Society for the Study of Vascular Anomalies, including clinical manifestations and magnetic resonance imaging (MRI). The radiologic criterion was a mass presenting a high signal on T2-weighted fat suppression (T2-W FS) sequences, with wide venous pools confirmed by contrast-enhanced magnetic resonance imaging (MRI). The following data were collected for these patients: sex; age; location; symptoms and signs; sclerosant; radiological and clinical outcome; complications; and changes in tracheal lumens. The clinical course of the patients is summarized in Table 2. This retrospective study was approved by the Ninth People’s Hospital Committee on Clinical Investigation as part of blanket institutional review board approval for the Vascular Anomalies Center.

### 2.2. Patient Treatment Protocols

#### 2.2.1. Sclerotherapy Before Tracheotomy

Among the 35 patients, 16 suffered from VMs located in the anterior cervical area. To reduce the intraoperative bleeding, these patients received 2~4 sessions of percutaneous sclerotherapy for these lesions before undergoing tracheotomy. Direct puncture was performed using 21 G needles. The double-needle technique was used in the case of the extravasation of sclerosants. The maximum dose in a single procedure was 0.2 mL/kg for absolute alcohol and 30 mL for foam sclerosing.

#### 2.2.2. Tracheotomy

**Figure 1:**
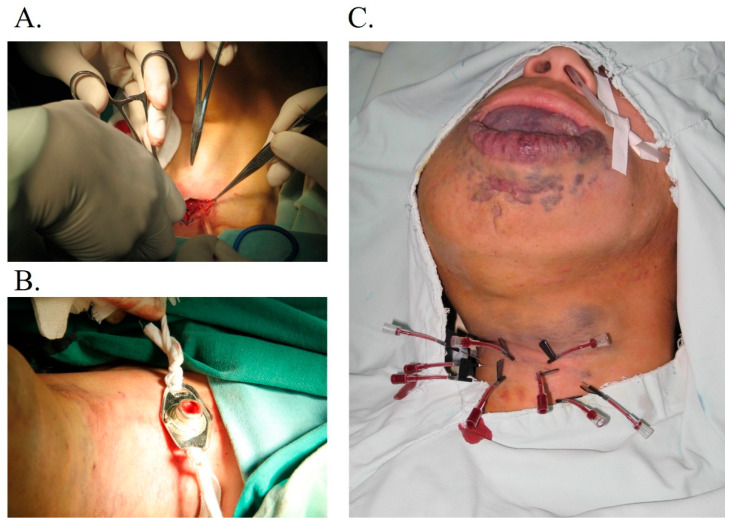
Schematic diagram of tracheostomy and sclerotherapy for extensive head and neck venous malformations. (**A**,**B**) Tracheostomy: A 2 cm horizontal incision was made 2 cm inferior to the cricoid cartilage. (**C**) Percutaneous ethanol sclerotherapy procedure: Two or more needles were punctured in the same VM cavity. Once the blood returned, absolute alcohol was injected, followed by 0.25% lidocaine.

The other 19 patients underwent the tracheotomy directly. Tracheotomy was performed in the operating room by the otolaryngologist under general or local anesthesia (Figure 1). Patients were prepped and draped in the standard fashion for tracheostomy. The cervical tissue 2 cm inferior to the cricoid cartilage was infiltrated with lidocaine 1% to epinephrine 1:100,000. A 2 cm horizontal incision was made in the region of the injected tissue. The superficial fascia and strap muscles were retracted laterally to expose the cricoid cartilage and thyroid gland. The thyroid was retracted either superiorly or inferiorly, or the isthmus ligated, depending on individual anatomy. A horizontal incision was made between the second and third tracheal rings, and an inferiorly based tracheal flap was sutured to the skin. The endotracheal tube was removed, and the tracheostomy tube was inserted under direct visualization. After cuff inflation, confirmation of placement was registered by auscultating the lung fields and viewing tidal volumes on the respirator. The tracheostomy faceplate was sutured to the skin with 2–0 silk sutures, and a twill tracheostomy tie was placed. Any complications were noted, including pneumothorax, wound problems, tube displacement, or blockage, accidental decannulation, laryngeal/subglottic stenosis, malacia, and tracheocutaneous fistula.

#### 2.2.3. Sclerotherapy After Tracheotomy

**Figure 2:**
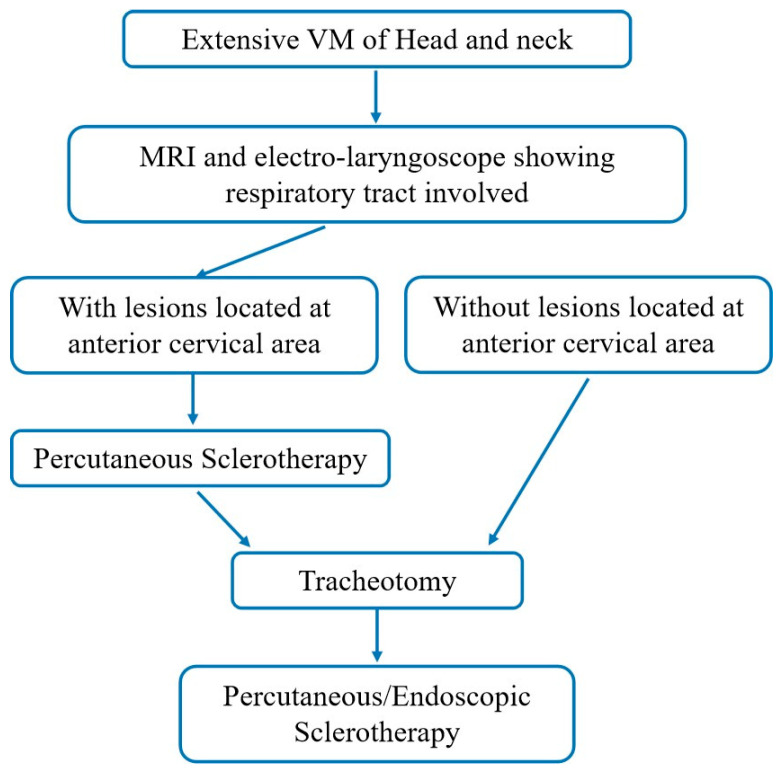
Flowchart of the surgical procedure.

Percutaneous sclerotherapy was performed under general anesthesia for pediatric patients or using intralesional lidocaine anesthesia in adult patients. A sterile field was created, and a direct puncture was performed through normal skin with a 21-gauge intravenous cannula. Once venous blood returned, contrast (Ultravist 300, Schering, Baver Leverkusen, Germany) was injected to opacify the malformation and to evaluate the resistance time of the contrast and the connection between the VM and the systemic circulation. If the extent of the lesion shown under fluoroscopy was significantly smaller than that demonstrated on MRI, more punctures were required in the same session. The treatment algorithm is concluded in Figure 2.

For lesions located above the tongue root, sclerotherapy was performed under a suspension laryngoscope. Among the patients, 27 received bleomycin-polidocanol foam (BPF) sclerotherapy prepared by dissolving bleomycin powder (15 mg, 15,000 IU; Nippon Kayaku, Tokyo, Japen) in 4 mL of 3% polidocanol solution (Aethoxysklerol; Kreussler Pharma, Wiesbaden, German). The sclerosant foam was generated using the Tessari method with two interconnected 5 mL syringes, employing a 4:1 air-to-liquid ratio. The mixture underwent emulsification through 20 rapid pumping cycles between the syringes. Ten patients were treated with absolute ethanol administered at a standardized dosage of 0.2 mL/kg. The tracheotomy tube was removed immediately after the treatment finished.

This staged treatment strategy, comprising prior sclerotherapy for anterior cervical lesion, followed by tracheostomy to establish a secure airway condition, and culminating in postoperative sclerotherapy to eliminate residual lesion, was designated as the ACSTS strategy.

### 2.3. Evaluation

Follow-up clinical data were collected from patients’ medical records. Clinical results were classified as worse, unchanged, or better. MRI findings before and after sclerotherapy were retrospectively reviewed by a radiologist and were classified as apparent cure, near-normal appearance, marked improvement but with residual malformations, minor improvement, and no change or worse. Patients’ clinical response was evaluated by physicians and was categorized as complete relief, significant response, partial response, and no change. In addition, patients’ evaluations of improvement in QoL were assessed through questionnaires during follow-up and classified into the following categories: excellent improvement, good improvement, moderate improvement, and no changes.

Complications were defined as minor or major. Minor complications included skin damage (such as skin blistering, skin ulcer, skin necrosis, cutaneous fistula, and pigmentation), nerve damage, muscle fibrosis, and transient hemoglobinuria. Major complications included tissue necrosis, pulmonary embolism, cardiovascular collapse, and even death.

### 2.4. Statistics

Descriptive statistics were used to analyze patients’ baseline characteristics. Continuous variables were assessed for normality using the Shapiro–Wilk test, with normally distributed data presented as mean ± standard deviation and non-normally distributed data described by median (interquartile range, IQR). Categorical variables were expressed as frequency (percentage). Spearman’s rank correlation analysis was employed to examine the strength and direction of correlations between variables. All statistical analyses were performed using SPSS 26.0 software, with statistical significance set at *p* < 0.05 (two-tailed).

## 3. Results

This study enrolled 35 patients, including 15 females (42.86%) and 20 males (57.14%), with a median age of 20 years (ranging from 2 to 76; IQR 21.57). Of all the 35 patients, 17 (48.57%) were younger than 18 years. The most common symptom was swelling (94.28%, 33/35), followed by snoring (57.14%, 20/35), lesion-associated pain (22.86%, 8/35), dyspnea (22.86%, 8/35), and hemorrhage (8.57%, 3/35).

Nineteen patients underwent a direct tracheotomy and sixteen patients received sclerotherapy at the incision area before tracheotomy. A total of 225 sclerotherapy sessions were performed in the 35 patients, with a median of 4.58 sessions (IQR, 4.12). The median tracheostomy cannulation duration was 13 months (IQR, 19.13).

After a median follow-up of 60 months (IQR, 102.25), postprocedural MRI demonstrated a volume reduction in the treated lesions in all 35 patients, including cure in 13 patients (37.14%) and near-normal appearance in 13 patients (37.14%), while 9 patients (25.72%) experienced a marked improvement but still had residual malformations. The clinical outcome is summarized in Table 3.

**Figure 3:**
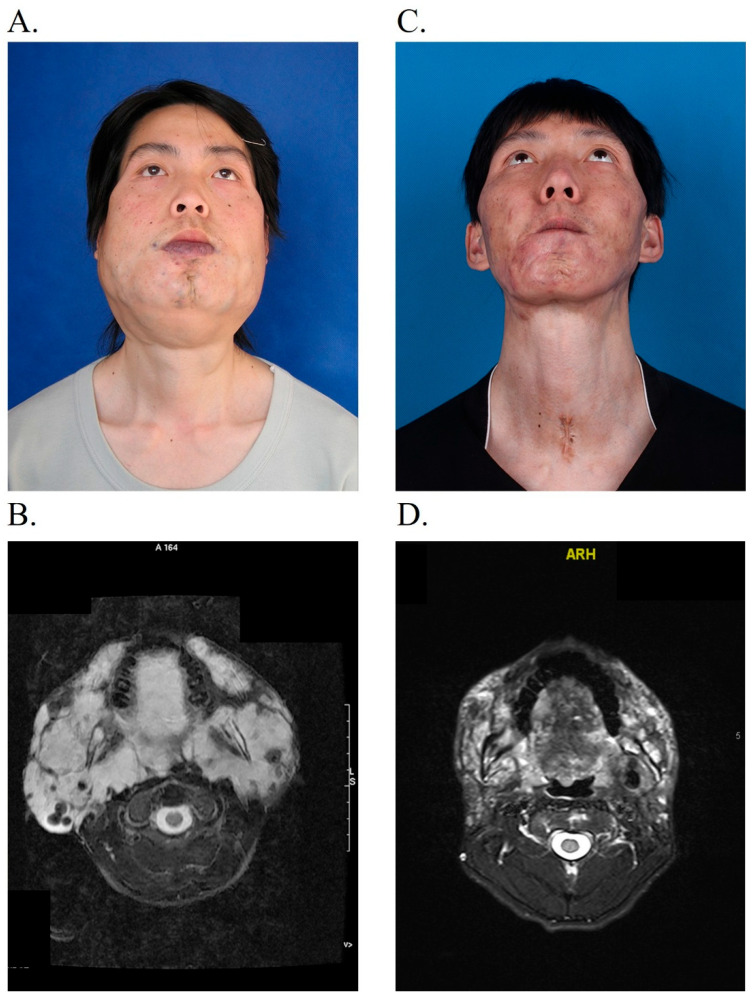
A 22-year-old man with a huge venous malformation in his face and neck. (**A**) The patient had his face, neck, and respiratory tract surrounded by tissue affected by VM. (**B**) MRI images show the lesion located in the surrounding tissue of the respiratory tract. The parapharyngeal space, tongue, and soft palate are involved. (**C**,**D**) Thirty-eight months later, after the tracheotomy tube was removed and the sclerotherapy was finished. Both the volume and extent of VM were evidently diminished.

**Figure 4:**
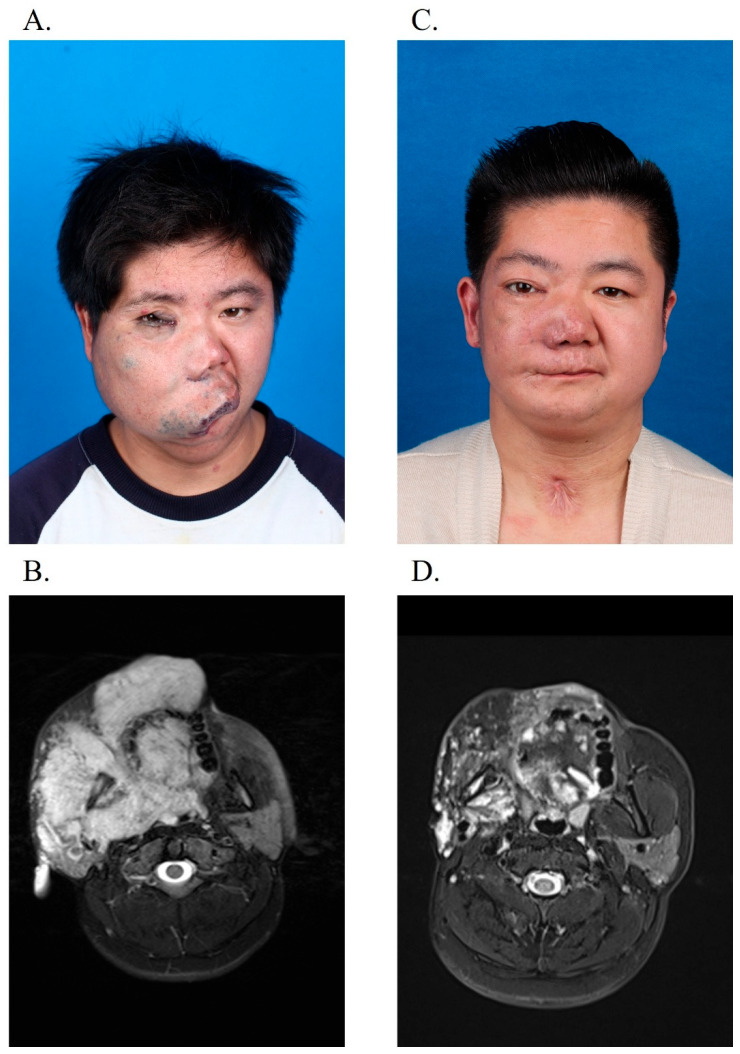
A 23-year-old male suffered from venous malformation involving the right face and neck. (**A**) Extensive venous malformations involved the right hemifacial and cervical regions. (**B**) MRI confirmed lesion extension into peripharyngeal tissues, encompassing the hypopharynx and tongue base. (**C**,**D**) Post-sclerotherapy and tracheostomy decannulation, 49-month follow-up revealed marked lesion regression.

In total, clinical response was reported in 33 (94.29%) cases. Among them, 6 patients (17.14%) achieved complete relief, 20 patients (57.14%) had a significant response, and partial response was seen in 7 cases (20%). Two patients exhibited no clinical response. No patients presented a worse change. Figure 3 and Figure 4 show the clinical improvements of the patients.

Furthermore, the patients described improvements in QoL. Twenty-three patients (65.71%) reported excellent improvement in QoL, eight patients (22.86%) experienced good QoL enhancement, and four patients (11.43%) showed moderate QoL improvement. Correlation analysis revealed a statistically significant correlation between overall clinical improvement and QoL enhancement (*p* = 0.003). However, no significant association between post-treatment MRI grading and clinical improvement (*p* = 0.703) or QoL outcomes (*p* = 0.908) was observed.

**Figure 5:**
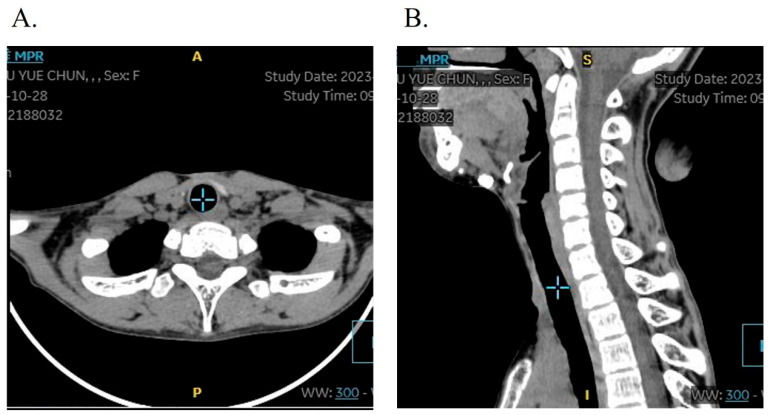
Follow-up CT of the tracheal tract in axial view (**A**) and sagittal view (**B**). There are no obvious structural changes to the trachea lumens.

Tracheotomy-associated complications were observed in three patients (8.57%, 3/35), comprising incision site tissue hyperplasia in all three cases (8.57%) and tracheal stenosis in one case (2.86%) who presented with mild tracheal softening and stenosis, demonstrating difficulty in decannulation after tracheostomy. A T-tube was implanted via electronic bronchoscopy, with postoperative follow-up confirming restoration of normal respiratory function and voice production. The follow-up CT showed a normal trachea structure without airway stenosis. Crucially, all patients underwent uneventful decannulation, with no instances of severe complications such as wound dehiscence or respiratory infections. Follow-up CT showed no obvious structural changes in the tracheal lumens (Figure 5).

In the sclerotherapy cohort, three patients (8.57%, 3/35) experienced self-limiting minor adverse events: cutaneous ulceration (5.71%, 2/35) and transient facial nerve palsy (5.71%, 2/35). The patients with cutaneous ulceration resolved completely without therapeutic intervention. For the two patients with facial nerve palsy, we prescribed mecobalamin tablets (Methycobal), a commonly used neurotrophic agent, at a dose of 0.5 mg to be taken three times daily. All patients recovered well.

Notably, no severe complications—specifically skin necrosis, anaphylaxis, thromboembolic phenomena, or persistent neurological impairment—were documented throughout the study duration.

## 4. Discussion

VM is a common vascular malformation and consists of abnormal veins that can be localized or can diffuse. The various treatment options for VM include sclerotherapy, surgical excision, laser therapy, and radiofrequency ablation [10].

In principle, an individualized treatment modality should be designed according to the location, size, and extent of the lesion; the speed of venous drainage; and technical availabilities. Sclerotherapy is a well-established, minimally invasive treatment for VM. However, extensive lesions located in the cervicofacial region frequently involve multiple tissues from skin to mucosa and may cause serious complications, such as bleeding, dyspnea, and dysphagia [11]. Therefore, a comprehensive multidisciplinary treatment is recommended for these challenging venous malformations. Notably, no systematic therapeutic strategies have been established for patients with airway-involving lesions, presenting a significant challenge in perioperative management. We described a treatment algorithm for head and neck VMs involving the upper respiratory tract. MRI and an electronic laryngoscope were necessary tests after the patients’ first visit. A multidisciplinary team, including anesthesia, ENT, and vascular specialists, created a comprehensive treatment algorithm for these challenging venous malformations.

Many different sclerosing agents are used to control venous malformation. As the most commonly utilized sclerosing agent, absolute ethanol is regarded as the gold standard due to its superior efficacy. Clinical evidence documents response rates ranging from 75% to 96%, accompanied by the lowest recurrence rates among currently available sclerosants [8]. Our previous investigations have demonstrated complete symptomatic improvement in all treated patients (100%), with an excellent and good response rate reaching 94.6%. Notably, no severe complications were documented, while minor complications occurred in 18.2% of cases, including skin hyperpigmentation, fever, and blisters. Notably, swelling occurred in all patients [7].

Swelling is a common side-effect and may lead to severe consequences when VM appears in the surrounding soft tissue of the airway. It can aggravate the airway obstruction symptoms. Once the respiratory tract is affected, the swelling may have a severe impact on respiratory function and may even lead to death due to suffocation [12]. Controlling swelling and airway-related complications during sclerotherapy while optimizing clinical efficacy has been a long-standing technical challenge. Once the swelling occurs, sometimes it is difficult for the patient to breathe spontaneously, and an emergency airway rescue is needed. Sun and his partners reported a case of severe airway problems of VM caused by swelling in the oral and maxillofacial region. By using the esophageal airway, they maintain the patient’s ability to breathe spontaneously. But before the esophageal airway was built, different methods of dealing with difficult airways had been used and failed [13]. Scott and his partners reported a life-threatening pharyngeal condition in a 68-year-old VM patient. After finishing a sclerotherapy, he experienced severe edema of the tongue and posterior pharyngeal wall, which caused constriction of his airway. The patient was intubated, and the edema had resolved 3 days later [14]. This issue is not reserved for VM-related therapies. Treatments for other diseases in which the airway is affected face the same problem: how to prevent airway obstruction caused by swelling. Many doctors have described their methods. Batra and his partner introduced the use of a laryngeal mask airway to rescue a patient with an arteriovenous malformation causing severe bleeding and swelling [15]. Harsha reported a case of airway obstruction caused by swelling after steroids were used to treat lymphovenous malformation. After the patient underwent a tracheotomy, the clinical symptoms improved. But these reports did not describe a safe, workable, and normalized method for dealing with the swelling caused by ethanol sclerotherapy in huge VM with respiratory tract involvement [16].

Tracheotomy is a routinely performed and widely accepted procedure for ventilator and airway obstruction. It is well tolerated and considered to be a safe procedure. During sclerotherapy, tracheotomy is usually regarded as the last rescue measure for airway obstruction [17]. For this reason, we suggested that an early-stage tracheotomy might be a good choice in patients whose upper respiratory tract is involved with VMs, particularly in patients with extensive lesions involving the laryngopharynx or tongue base, or the lateral walls of the oropharynx bilaterally. For patients whose lesions involve the anterior cervical area (2 cm inferior to the cricoid cartilage), tracheotomy was performed by ENT after sclerotherapy for these lesions in case of intraoperative bleeding. If the anterior cervical area was not involved, patients underwent direct tracheotomy. Subsequently, sclerotherapy was performed among these patients. After a period of observation and efficacy evaluation, decannulation was performed.

Tracheostomy-associated complications are prevalent, with critically ill patients demonstrating elevated risk profiles. Strober et al. documented that 10.3% of adult patients experienced at least one tracheostomy-related complication within 90 postoperative days. The incidence spectrum included stomal dysfunction (3.2%), hemorrhage (1.6%), localized infection (0.9%), and tracheoesophageal fistula formation (0.2%). Additional mechanical complications—notably tube obstruction and accidental dislodgement—occurred in 5.6% of cases [18]. Tracheal stenosis represents a frequent tracheostomy-associated complication, occurring in 1.85% of patients, according to Goldenberg et al. Their analysis further quantified procedural risks: tube obstruction (0.35%), pneumothorax (0.26%), subcutaneous emphysema (0.08%), and glottic stenosis (0.08%) [19]. In our study, tracheostomy was well tolerated without significant complications. There was also no serious discomfort during the intraoperative, early postoperative, and late postoperative stages of tracheotomy, except for one case of tracheal stenosis. Therefore, it can be postulated that an early tracheotomy before systemic sclerotherapy might be a feasible and safe choice in upper tracheal tract-related VM.

Our study was a preliminary design, which involved some inherent flaws. As this was a retrospective study, the data we collected may carry inherent selection and information biases. Additionally, insufficient CT-based assessments could lead to the potential missed recording of complications. Randomized, controlled trials with enough patients, and with prospective and long-term follow-up, will be necessary to determine the benefits of tracheotomy in avoiding airway obstruction symptoms caused by swelling.

This study confirms the safety and efficacy of tracheostomy-based sclerotherapy for upper airway VMs, and is particularly necessary in those affecting critical anatomical zones (laryngopharynx, tongue base, bilateral pharyngeal walls). For high-risk cases requiring airway protection, we propose a triphasic protocol called the ACSTS sequence, which begins with preprocedural sclerotherapy for lesion reduction, followed by tracheostomy for secured ventilation, and definitive sclerotherapy for residual ablation.

## Figures and Tables

**Table 1:** Clinical characteristics.

Clinical Characteristics	
Gender	
Male	57.14%, 20/35
Female	42.86%, 15/35
Age (years) (median, IQR)	20, 22
Symptom	
Swelling	94.28%, 33/35
Snoring	57.14%, 20/35
Lesion-associated pain	22.86%, 8/35
Dyspnea	22.86%, 8/35
Hemorrhage	8.57%, 3/35

**Table 2:** Clinical course.

Clinical Course	
Sclerotherapy before tracheotomy	
Yes	45.71%, 16/35
No	54.29%, 19/35
Tracheotomy cannulation duration (months) (median, IQR)	13, 19.13
Sclerotherapy sessions (median, IQR)	4.58, 4.12
Sclerosants	
Pingyangmycin-polidocanol foam	40%, 14/35
Bleomycin-polidocanol foam	42.86%, 15/35
Absolute ethanol	28.57%, 10/35
Follow-up (months) (median, IQR)	60, 102.25

**Table 3:** Clinical outcome.

Clinical Outcome	
MRI grades	
Cured	37.14%, 13/35
Nearly normal appearance	37.14%, 13/35
Marked improvement, but still with residual malformations	25.72%, 9/35
Minor improvement	0, 0/35
No change or worse	0, 0/35
Clinical response	
Complete relief	17.14%, 6/35
Significant response	57.14%, 20/35
Partial response	20%, 7/35
No changes	5.71%, 2/35
QoL improvement	
Excellent improvement	65.71%, 23/35
Good improvement	22.86%, 8/35
Moderate improvement	11.43%, 4/35
No changes	0, 0/35
Tracheotomy complications	8.57%, 3/35
Incision site tissue hyperplasia	8.57%, 3/35
Tracheal stenosis	2.86%, 1/35
Sclerotherapy complications	8.57%, 3/35
Cutaneous ulceration	5.71%, 2/35
Transient facial nerve palsy	5.71%, 2/35

## Data Availability

Our data are available from the corresponding author upon request.
